# Specialist nurses’ perceptions of inviting patients to participate in clinical research studies: a qualitative descriptive study of barriers and facilitators

**DOI:** 10.1186/s12874-016-0204-5

**Published:** 2016-08-11

**Authors:** Caroline French, Charitini Stavropoulou

**Affiliations:** 1 Centre for Primary Care and Public Health, Blizard Institute, Queen Mary University London, 4 Newark Street, London, E1 2AT UK; 2 School of Health Sciences, City University London, Northampton Square, London, EC1V 0HB UK

**Keywords:** Clinical nurse specialist, Patient selection, Research participant recruitment, Qualitative research, Research conduct

## Abstract

**Background:**

Increasing the number of patients participating in research studies is a current priority in the National Health Service (NHS) in the United Kingdom. The role of specialist nurses in inviting patients to participate is important, yet little is known about their experiences of doing so. The aim of this study was to explore the perceptions of barriers and facilitators held by specialist nurses with experience of inviting adult NHS patients to a wide variety of research studies.

**Methods:**

A cross-sectional qualitative descriptive study was conducted between March and July 2015. Participants were 12 specialist nurses representing 7 different clinical specialties and 7 different NHS Trusts. We collected data using individual semi-structured interviews, and analysed transcripts using the Framework method to inductively gain a descriptive overview of barriers and facilitators.

**Results:**

Barriers and facilitators were complex and interdependent. Perceptions varied among individuals, however barriers and facilitators centred on five main themes: i) assessing patient suitability, ii) teamwork, iii) valuing research, iv) the invitation process and v) understanding the study. Facilitators to inviting patients to participate in research often stemmed from specialist nurses’ attitudes, skills and experience. Positive research cultures, effective teamwork and strong relationships between research and clinical teams at the local clinical team level were similarly important. Barriers were reported when specialist nurses felt they were providing patients with insufficient information during the invitation process, and when specialist nurses felt they did not understand studies to their satisfaction.

**Conclusion:**

Our study offers several new insights regarding the role of specialist nurses in recruiting patients for research. It shows that strong local research culture and teamwork overcome some wider organisational and workload barriers reported in previous studies. In addition, and in contrast to common practice, our findings suggest research teams may benefit from individualising study training and invitation procedures to specialist nurses’ preferences and requirements. Findings provide a basis for reflection on practice for specialist nurses, research teams, policymakers, and all with an interest in increasing patient participation in research.

**Electronic supplementary material:**

The online version of this article (doi:10.1186/s12874-016-0204-5) contains supplementary material, which is available to authorized users.

## Background

The potential of high-quality clinical research to bring widespread population health and economic benefits is globally recognised [1]. In the UK, the conduct of clinical research is a core role of the NHS [2], and *The NHS Constitution for England* pledges to inform all patients about opportunities for involvement with suitable research studies [3]. In this context healthcare professionals play a vital role in clinical research, linking researchers and patients by delivering research invitations in the course of providing clinical care. Yet it has been recognised that, having agreed to support recruitment, healthcare professionals do not always then invite all potentially eligible patients to studies [4]. As a result, the number and diversity of patients invited to research studies may be limited, potentially leading to ethical and methodological problems associated with selection bias and below-target recruitment [4, 5].

A number of previous studies have investigated barriers and facilitators to a variety of healthcare professionals inviting patients to participate in research, including general practitioners [6–8], practice nurses [8, 9], pharmacists [10], and mental health care coordinators [11, 12]. While a wide range of factors affecting recruitment have been identified, many of these studies were conducted in relation to recruitment to specific studies, often a single randomized controlled trial (RCT). As often acknowledged by authors, this may restrict the breadth of barriers and facilitators identified. Recent syntheses of qualitative findings have nonetheless helped enhance generalisability by identifying a number of common themes across such studies, including clinician engagement with research, perceptions of research interventions and workload [5, 13]. However, Fletcher and colleagues published their qualitative syntheses alongside a systematic review of interventions aiming to improve recruitment of patients by clinical staff to RCTs [5]. This highlighted that ‘common sense’ interventions often had little success, and that greater understanding of barriers and facilitators to clinical staff inviting patients to research is required [5].

Previous studies investigating barriers and faciliators to inviting patients to specific studies have been useful in understanding organizational and context specific issues, however they have certain limitations, including potential researcher bias and the possibility of participants providing socially desirable responses [5, 14]. There is therefore a gap in the literature regarding healthcare professionals’ perceptions of inviting patients to research as a general concept, rather than in relation to a specific study. Investigating general perceptions may bring the advantages of researcher independence and unrestricted sample size, as well as a wider range of perspectives, which may provide new insights. There are some examples of previous studies that have taken this approach, although with more specific foci. For example, Nurmi and colleagues explored Finnish hospital nurses’ perceptions of ethical issues surrounding inviting patients to research, interviewing nurse leaders rather than nurses doing the inviting [15]. They found positive and timely collaborations with researchers, a positive research culture and opportunity for quiet moments to discuss research with patients to be facilitators to delivering invitations.

The aim of this study was to explore specialist nurses’ perceptions of barriers and facilitators to inviting adult patients to participate in research studies during clinical care encounters. Specialist nurses are often well-placed to invite patients to participate in research [16], and therefore increased understanding of barriers and facilitators they may encounter is desirable. Although some previous research has explored experiences of specialist nurses of involvement in trials [17, 18], those specialist nurses had responsibilities and involvement in the whole trial, rather than solely inviting patients during their clinical roles.

**Fig. 1:**
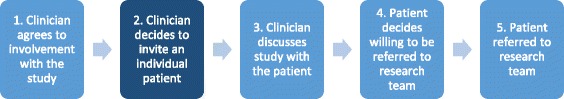
Process of a patient being referred to a research team by a clinician (adapted from Frayne and colleagues [19])

Frayne and colleagues conceptualise a process by which a patient may be referred to a research study when the initial invitation to participate is delivered by a healthcare professional in the clinical setting [19], illustrated in Fig. 1. Our study focuses on the second stage of this process.

## Methods

### Methodology and rationale

We employed a qualitative descriptive design to explore specialist nurses’ perceptions. Qualitative description is well-suited to understanding perceptions of stakeholders regarding practice issues by seeking to generate detailed descriptions of phenomena as framed by participants [20]. An ontological stance of subtle realism as described by Hammersley [21] was adopted, which accepts that social phenomena can be studied and known, however perceptions of these phenomena vary between individuals. Complementary to this stance, an epistemological standpoint of empathic neutrality was adopted, in which it is acknowledged that researchers inevitably influence research findings, however this influence should be recognised and minimised [22].

### Conceptual framework

**Fig. 2:**
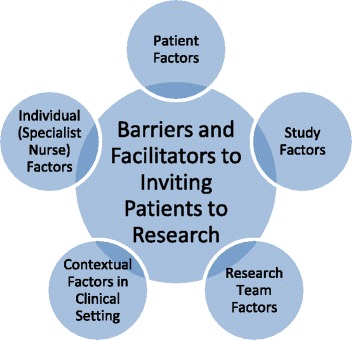
Conceptual framework

To our knowledge there were no established conceptual frameworks that could be applied to describe barriers and facilitators to inviting patients to studies. We therefore developed a simple conceptual framework from a review of previous research studies that have addressed similar research questions. The conceptual framework (Fig. 2) assumes the theoretical standpoint that barriers and facilitators may exist at five levels, and these may interact to influence the outcome of a patient being invited to a study. As well as clarifying the theoretical basis for this study, we used the conceptual framework to inform data collection and place findings in context of existing knowledge. Our approach was primarily inductive and we did not intend the conceptual framework to restrict findings, with both data collection and analysis methods allowing new concepts to emerge.

### Sampling

Specialist nurses working in general adult acute care or community settings with current or recent (within 6 months) experience of inviting adult NHS patients to participate in research studies were eligible for the study. The term specialist nurse included a variety of job titles, such as clinical nurse specialist or nurse practitioner, the unifying feature of the role being practising in a specialist clinical role as an independent practitioner with a caseload of patients. The focus of the study was on inviting patients to participate as an add-on to delivering clinical care, and therefore nurses with dedicated study recruitment roles (such as research nurses, or those with time bought by researchers) were excluded. Specialist nurses who were responsible for delivering research interventions within the same trial were also excluded.

With purposive sampling we aimed to recruit specialist nurses from a wide variety of clinical specialties and NHS Trusts. Both of these variables have been identified as possible influences to clinician attitudes and experiences of research [23, 24]. Additionally, it was anticipated that participants who varied in these characteristics would inevitably also have experience of inviting patients to different research studies. However, while we purposively invited specialist nurses from a range of clinical specialties and NHS Trusts, we also employed convenience sampling to recruit participants on a first-come first-served basis. Given the time and resource constraints of this study, this was a pragmatic alternative to purposively selecting participants from a pool of respondents.

Other factors such as attitudes to research [25] and educational qualifications [26] have also been identified as influential to perceptions of recruiting patients to studies. Demographic data were collected on these factors, however it was not possible to prospectively purposefully invite nurses who varied on these characteristics.

### Ethics

The study received ethical approval from City University School of Health Sciences Ethics Approval Committee, and research governance approval from the NHS Trusts used to recruit participants. All participants gave written informed consent, and all transcripts and reports were anonymised.

### Recruitment

We circulated email invitations to specialist nurses via local collaborators in three NHS Trusts and three clinical specialty networks. These recruitment sites were selected by convenience as one of the authors (CF) had existing contacts in these sites to facilitate the process of gaining approvals and circulating invitations.

### Data collection

We used face-to-face individual semi-structured interviews to collect qualitative data between March and July 2015. Interviews began with broad questions relating to job role and experience and opinions of research, and then moved to questions about barriers and facilitators to inviting patients to studies. We based main questions on the five levels within the conceptual framework, also asking participants about any issues of relevance not covered by these questions. The interview topic guide can be found in Additional file 1. Our interview structure was flexible and questions open ended, in order to allow participants to determine issues of importance. We audio-recorded interviews in participants’ preferred locations (away from the clinical environment), and collected demographic data using a questionnaire prior to interview commencement.

### Data analysis

We undertook a thematic analysis of the qualitative data using the Framework method [27], with the aim of inductively gaining a descriptive overview of barriers and facilitators. Briefly, we developed an initial data management framework by inductively coding four transcripts, selected to represent different specialties and Trusts. We then organized all codes into main categories and subcategories, and applied this initial data management framework to index the remaining transcripts. We created matrices using Microsoft Excel and populated these with data from the indexed transcripts, with many pieces of data relevant to more than one cell. The process of using the data management framework to populate the matrix enabled a deep level of immersion and familiarity with the data and also highlighted many areas of overlap between themes. From this process, and reviewing the matrix and transcripts several times, we noted several crosscutting main themes. We then extracted relevant pieces of data from across the dataset for each crosscutting theme, and organized these to create subthemes. We produced a final narrative analysis from these crosscutting themes and subthemes, with the matrix providing an easily accessible reference to compare the differing views of participants and check original data. After producing the final themes, subthemes and results narrative, we reviewed all transcripts and matrices to check the final results reflected the overall feel of the original data.

### Rigour and reflexivity

CF conducted all of the interviews as a full-time Masters’ student, seconded from a role as a research nurse. Aside from arranging interviews, she had no previous relationships with 11 of the participants prior to interviews, but had worked indirectly with one. She had no history of connections with most of the studies or research teams participants discussed, although had previous peripheral involvement as a research nurse with a few studies mentioned. She informed participants during consent that the study was being undertaken for a Masters’ dissertation and that motivations to undertake the research stemmed from research nurse experience.

Before data collection commenced, CF piloted interviews with colleagues, who provided feedback on her interview style. This enabled her to refine the structure and technique of the interview before collecting data. In addition, and throughout the study, CF had regular meeting with CS, during which they discussed overall progress and issues that arose from the interviews already conducted. CF used reflexivity to develop technical and interpersonal skills over the course of the study in order to encourage participants to give open and detailed responses.

In order to identify and address potential researcher bias, CF noted her preconceptions and reflected on motivations for doing the research prior to commencing data collection. She used reflexivity throughout the data collection and analysis processes to identify potential influences of her preconceptions and motivations. Two transcripts were independently coded by a CS, with codes discussed and agreed.

## Results

### Participant characteristics

Twelve specialist nurses participated, representing 7 different clinical specialties and 7 different NHS Trusts. A further four initially expressed interest however then either left post (1) or did not respond to further communication (3). Demographic data are presented in Table 1. Qualitative data showed that participants had a wide range of experience of inviting patients to different types of studies, from simple questionnaire surveys to early-phase pharmacological RCTs. Many had some wider experience of research, for example having carried out their own research projects, or having previous experience as a research nurse.

**Table 1:** Participant characteristics

	Number of participants (%) *N* = 12
Age	
31–40	2 (17)
41–50	7 (59)
51–60	3 (25)
Clinical specialty	
Cancer	3 (25)
Stroke	3 (25)
Vascular surgery	2 (17)
Cardiac prevention and rehabilitation	1 (8)
HIV	1 (8)
Parkinson’s disease	1 (8)
Pulmonary hypertension	1 (8)
Employing NHS Trust (type)	
Acute teaching hospital	9 (75)
Community trust	2 (17)
District general hospital	1 (8)
Gender	
Female	12 (100)
Male	0 (0)
Highest educational qualification	
Registered nurse (non-degree)	2 (17)
Bachelor’s degree	2 (17)
Postgraduate certificate	1 (8)
Postgraduate diploma	3 (25)
Master’s degree	4 (33)

### Themes

We identified five main themes of barriers and facilitators, with a total of 12 subthemes.

#### Theme 1: Assessing patient suitability

All participants discussed considering individual patient suitability to be invited to participate in research, additional to each particular study’s inclusion criteria. The following subthemes reflect different ways in which participants assessed patient suitability and dealt with any concerns.

##### Subtheme 1.1: Not inviting the patient because of anticipated negative impacts

Many participants discussed concerns that certain patients could be negatively affected by being invited to participate in research, for example if they were unwell, disabled or elderly. These anticipated negative impacts included negative emotions, such as fear or distress, or feeling overburdened with information. Some specialist nurses also highlighted that certain studies, such as those requiring multiple visits to a research site, may place a strain on some patients.

Some participants had personal and professional ethical views that they should protect these patients from such perceived negative impacts by not inviting them.

*I would not say anything about any research if that particular person was having a bad time at that point in their stroke pathway because I don’t think it’s fair to add another thought process to their recovery.* (003)

Many of these participants discussed weighing up different factors before deciding whether or not to approach eligible patients, including consulting with other members of the healthcare team.

Some specialist nurses mentioned that they may consider certain patients’ participation as potentially detrimental to the research study, for example patients unable to reliably recall information. This could similarly be a barrier to inviting them.

##### Subtheme 1.2: Inviting the patient because of perceived positive impacts

Sometimes anticipated positive impacts for the patient of being invited to a study, such as feeling included or excited, were the most important considerations. Some specialist nurses felt not inviting patients was paternalistic, and could deny them beneficial treatments or experiences. Prior personal and professional positive experiences of research, including having been a research participant, or having previously made incorrect assumptions about patient wishes, contributed to these viewpoints.

An awareness of positive impacts could however raise barriers. Several participants said they were cautious about inviting patients unless they were confident they would be eligible for the study, as they did not want to raise false hopes. Therefore, they needed the research team to provide them with clear eligibility criteria. One participant reflected that she may invite patients more enthusiastically who she felt most likely to benefit from the research intervention.

##### Subtheme 1.3: Inviting the patient despite anticipating negative impacts

Some specialist nurses said that they tried to invite all eligible patients to studies, despite sometimes having concerns about individual suitability. This was because they felt they had a duty to researchers to do their best to help recruit, or their understanding of the research process meant that they appreciated the negative consequences of selection bias. Nevertheless, two reflected that their research knowledge made them less likely to invite patients who they perceived as potentially detrimental to the research study.

Some participants, who invited patients despite anticipating negative impacts reflected they sometimes felt uncomfortable doing so, and acknowledged they may subtly modify their approach.

*I don’t necessarily like the ones where it inconveniences the patients, you’re asking them to come in for lots of extra appointments, things like that where I feel like, gosh, that’s a bit unfair. So I probably go into those ones with a little bit less enthusiasm…* (005)

However, participants described also several ways in which they could avoid or minimise perceived negative impacts on patients, and could therefore overcome reservations about inviting them. It was helpful if, prior to study commencement and during the study, specialist nurses discussed and resolved concerns with the research team.

*Usually we’ve had discussions as a team with researchers that are coming in, and thrashed out some of the issues we might have been concerned about. That we’ve resolved most of the issues and so at that point we’re happy to be involved in approaching or introducing patients to what a research team are doing*. (012)

Some specialist nurses raised that they considered themselves well placed to invite patients in a non-threatening and sensitive manner. This could mean being able to time the approach to when they perceived the patient to be in the best position to receive the information, or having sufficient time to spend with the patient to allay potential negative emotions. Having flexibility to manage their time and ongoing regular contact with patients made this possible. The supportive nurse-patient relationship also appeared to be very important. Many participants felt trust and rapport enabled them to feel comfortable their patients would not feel pressurised, and enabled them to anticipate and address negative emotions.

*Because if you trust me and we’ve built up a relationship where you feel safe and comfortable, then if I start talking to you about research and you feel safe and comfortable, even if you feel a little bit anxious about the idea of being experimented on…* (002)

Similarly, some participants felt that their clinical relationship and supportive roles made them feel comfortable to invite patients during times of distress, and to gently explore patients’ reasons for declining participation.

Several participants mentioned how they considered different clinical environments to be more or less conducive to inviting patients in a sensitive manner. Busy clinic and ward environments were felt to be barriers, while more relaxed environments such as patients’ homes and ward day rooms were considered more suitable.

#### Theme 2: Teamwork

The importance of teamwork was highlighted by the participants. This included teamwork both within their clinical teams and with research teams, as explained in the two subthemes below.

##### Subtheme 2.1: Teamwork within the clinical team

Several participants emphasised that engagement and motivation arose from a sense of working as a team with clinical colleagues to recruit patients. Conversely, personal motivation was more difficult to achieve when there was a sense members of the clinical team were not on board.

*I didn’t feel like the rest of the team had that as a priority and so it’s hard to do that when you’re the only one doing it*. (009)

Several participants gave examples of how clinical teamwork could have an impact on the patient experience of being invited to participate in research. They therefore felt happier to invite patients when their clinical team presented a unanimous and coordinated approach. A benefit of such an approach was avoiding negative impacts on patients resulting from receiving conflicting information.

*…these studies were there, they’re not mentioned by a doctor, we try and mention it, and it causes upset, well the doctor didn’t say that?* (010)

Some participants also described how clinical teamwork had led them to develop team recruitment strategies, such as measures to ensure all eligible patients were invited to studies.

##### Subtheme 2.2: Teamwork with the research team

A sense of teamwork with the research team was very important to promoting engagement with and ownership of the study. Several participants also felt this helped them to remember to invite patients and to motivate them to overcome organizational barriers such as workload. A sense of teamwork helped promote a sense of mutual collaboration, resulting in a desire to help research team members as colleagues.

This sense of teamwork could stem from research team members also being clinical team members, such as when the study had been within their team. If the research team was external it was helpful if they were accessible and approachable, maintained communication, and had a regular presence in the clinical area.

*So the fact that the research team know and put up with us…and are happy to keep asking us and don’t lose the plot with us helps…* (011)

Several participants explained that they wished to help researchers, however had found it difficult to sustain motivation when they perceived the research team as uncommunicative, unapproachable or inefficient. Participants described experiences of research team behaviours that had enhanced or detracted from this sense of teamwork.

*…the previous research nurse…understood that the best way to engage us to participate, the best way to get us onside and the best way to get optimum recruitment was to make everything as easy as it could be for us.* (002)

#### Theme 3: Valuing research

Specialist nurses’ favourable personal and professional opinions of research, and working in clinical environments with positive research cultures, were often facilitators for inviting patients to participate in studies.

##### Subtheme 3.1: Personal valuing and experience of research

All participants felt very positive about research, which they felt often motivated them to invite patients to studies. Their positive views usually stemmed from their own experiences, such as attending conferences, prior involvement in conducting research, or having themselves been research participants.

A key element of understanding research appeared to be seeing clearly the link between inviting patients to participate in studies and positive impacts on patient care and clinical practice. Some participants had first-hand experience of this and felt it was a very strong motivator to inviting patients to research.

*…I think you almost have to come through the cycle to see the impact of one of those studies and what it has now done, and how surgery has changed because of that study to fully see the benefit…* (005)

A barrier to inviting patients to studies was the time taken for research to bring about changes in practice. Some nurses felt that research teams could promote the value of research involvement by explaining how results of previous studies had led to recent practice developments. In addition, nurses who had helped in the recruitment process were often not involved in dissemination activities. This was seen as missed opportunity, as nurses did not then see the relevance of the study findings in building further collaborations and research.

##### Subtheme 3.2: Inviting patients to participate in research within the specialist nurse role

Many participants felt that inviting patients to participate in research was an important aspect of their clinical role. They related this both to benefits for individual patients of research participation, and to their role in contributing to improving future care for their patient population.

*…and eventually it’s going to come out and be for patients, which at the end of the day is what I’m here for anyway, and if I can help in that then I’m happy to do whatever.* (003)

Participants generally felt that certain aspects of their clinical roles, such as flexibility and autonomy, were conducive to inviting patients to studies. Nonetheless, some were restricted by time, workload and distractions within their clinical roles. The specialist nurses who raised these issues said that time or forgetfulness were rarely absolute barriers, however became more of a problem when compounded by other barriers such as lack of research team communication.

##### Subtheme 3.3: Research culture

The value placed upon research in the clinical setting could facilitate or obstruct inviting patients to participate in studies. Some participants mentioned the influence of organisational research culture, within their NHS Trust or clinical specialty. However, they spoke much more extensively about the influences of local research cultures, generally within their clinical teams, and several gave examples of their local culture strongly facilitating inviting patients in research. Participants generally felt these local cultures had developed from within over time, influenced by people within the team who were strongly research-oriented.

*…we’re very lucky in the sense that we’ve got a few professors knocking about, and they’re all really keen on research, but we’ve got some strong characters around them as well, like the research nurses are very strong character…and I’m not a mouse…so when you get people saying the same things…So there’s a network of influence there which I think has quite a powerful effect, but it doesn’t just happen overnight, it’s like a dripping tap effect*… (010)

#### Theme 4: A satisfactory invitation process

All participants discussed the methods by which they invited patients to participate in studies, and often had strong preferences for how this should occur. Barriers could therefore arise when the invitation process was unsatisfactory.

##### Subtheme 4.1: Convenience of the invitation process

For several participants, complex or time-consuming study invitation processes, such as needing to apply complex eligibility criteria, could be barriers to inviting patients. Conversely, a process that was simple, with the research team providing tools to facilitate the process such as checklists and flowcharts, was perceived as helpful. It was however important that any tools provided were in an appropriate format.

*One of the big thing’s that’s really good about them is they laminate them, because there’s nothing worse than a scruffy bit of paper that you’ve carried around in a folder for two years that just gets missed at the end*. (008)

##### Subtheme 4.2: Giving information as part of the invitation process

Many specialist nurses believed they should give patients a reasonable amount of information about studies alongside inviting them to participate, which could be more than they were expected to give by the research team. They generally also felt they should be able to answer patients’ questions. Giving the patient a small amount of information, with the research team then providing full information if the patient agreed to see them, was generally viewed negatively.

*…because you can’t just give them a form and say oh, off you go, have a look at that, it’s nice to talk to them a little bit first, yeah*. (004)

Some participants felt patients viewed it negatively when they were invited by somebody who could not explain the study properly, and were therefore less likely to agree to participate. This was important for some participants, who saw it their responsibility to present studies in a favourable light. Other participants felt differently however, sensing that patients did not mind being given incomplete information.

#### Theme 5: Understanding the study

As discussed in previous themes, it was important to participants to feel sufficiently informed about studies when inviting patients to participate. They had varying requirements, with some feeling it was a professional responsibility to have complete understanding, and others being happy with an overview. Whatever their individual requirements, most participants viewed having insufficient understanding negatively.

*I always feel a little bit miffed if I don’t have a full understanding of what’s involved, and what I’m asking them for, and what the outcome of it is going to be.* (007)

##### Subtheme 5.1 Research team interventions to increase understanding of the study

Research teams played a key role in enhancing participants’ level of understanding of the study, and therefore if their efforts were unsuccessful this could be a barrier to inviting patients to participate. Many specialist nurses said it was important for research teams to explain the study to them fully before they started approaching patients, with face-to-face contact being particularly helpful. Some participants however gave negative examples of research teams’ attempts to explain studies, such as boring presentations and overly complicated information.

*…if it hasn’t been sold to me well enough why would I then I suppose take on, so I definitely think the research team have got a huge role really*. (006)

Easy access to information throughout the study was a facilitator, and the location and format of this information was important. A research team member being embedded within the clinical team was perceived as particularly helpful, as it meant somebody was on hand to answer queries. Research teams being perceived as unapproachable could discourage seeking further information, clarification and reassurances.

Close relationships with the research team could also provide opportunities to enhance understanding.

*So a good working relationship with them certainly does help…we get involved, so we go across to the research centre sometimes and see how the patients, the clinical reasons why they’re having the research so that it’s not completely disjointed…* (008)

##### Subtheme 5.2 Specialist nurses’ ability to understand the study

Some participants gave examples of taking proactive measures to increase their own understanding of studies. These included reading through study information, accessing study websites or preparing questions to take to study initiation meetings. Nevertheless, some specialist nurses felt that lack of time and research experience could limit their abilities to do this.

Specialist nurses generally felt their understanding of the value of a study was very apparent when the topic related to a perceived current need of their patients. They particularly highlighted studies developed within their clinical team, or where they had been consulted or involved during the design of the study.

## Discussion

This study identifies that barriers and facilitators to specialist nurses inviting patients to participate in research are complex and interdependent. The five main themes show broad issues that appear to be important to specialist nurses, although individual views to the relative importance of barriers and facilitators across these themes varied. In order to contextualise these with previous research and consider implications for different stakeholders, the discussion is framed by the conceptual framework (Fig. 1).

### Patient factors

Findings support previous research identifying that concerns about individual patient suitability are often barriers to healthcare professionals inviting patients to studies [5, 7, 11–13, 26]. It is a common perception that such concerns of healthcare professionals are well-meaning but often misplaced, such ‘gatekeeping’ thus being potentially detrimental to both research success and patient autonomy [28, 29]. Donovan and colleagues [30] identified however that nurses may experience an uneasy role conflict between providing clinical care and inviting patients to participate in research. Our study supports this, as several participants expressed discomfort at broaching the subject of research if they considered it not in a patient’s best interests. This suggests, as recommended by Donovan and colleagues [30], that specialised training and support for clinicians involved in research recruitment are required. Training in Good Clinical Practice (GCP) principles provides information relating to the legalities of obtaining informed consent, however does not usually address gatekeeping and related issues.

Conversely, several specialist nurses felt patient factors should not be barriers to inviting patients, agreeing that this diminished patient autonomy. Additionally, and in common with previous findings [8], an understanding of potential benefits to patients could facilitate inviting research participation. This suggests that measures to highlight patient experiences of research are likely to be helpful, both by study teams and through broader initiatives. The *Research changed my life* campaign within the NHS [31], is an example of the latter, where patients across England share stories of how their lives were positively transformed by clinical research.

### Research team factors

Research team behaviours, particularly relating to communication, and the quality of relationships between specialist nurses and research teams, appeared to influence barriers and facilitators across all five themes. Some of the links between these behaviours and the subsequent creation of a barrier or facilitator appear fairly obvious, such as failure to communicate the aims of a study leading to non-comprehension. However specialist nurses also gave examples of how these behaviours could have more subtle effects, for example that making efforts to provide clear and simple information could convey a sense of teamwork and hence increase motivation. Many previous studies have similarly identified research team conduct and relationships with clinicians as important to recruitment success [5, 13]. These findings highlight that research teams should continuously reflect upon their behaviours, communication and relationships with clinicians in order to benefit from fruitful collaborations.

Participants were forthcoming with several examples of negative experiences of research team conduct. A notable feature of some previous studies is that researchers report making extensive efforts to communicate effectively with clinical teams, but still facing recruitment problems [12, 32]. This raises the possibility that clinicians may be reluctant or unmotivated to express dissatisfaction or misunderstandings. Research teams should therefore encourage clinical collaborators to express their needs and give feedback, and promote honest and open communication.

### Study factors

Previous research has identified that barriers to healthcare professionals inviting patients often result from concerns about research study interventions or randomisation processes [12, 13]. These issues were however generally much less contentious to specialist nurses in this study, which could partly be explained by interviews focusing on general rather than study-specific issues.

The most important study-related factor appeared to be the nature of the invitation process, that is, the process specified by researchers to identify, approach and refer a patient to their study. The finding that a barrier to inviting patients is an excessively long-winded invitation process is supported by the literature, with many healthcare professionals preferring simple and quick recruitment procedures [5]. Nonetheless, it was important to many of the specialist nurses in this study to give patients a reasonable amount of information about a study, and importantly, they were often willing and able to spend time doing so. Some previous studies have found that healthcare professionals require their time and effort over the entire invitation process to be minimised [6, 12]. However, our findings suggest it may be counterproductive for research teams to attempt to reduce burden on specialist nurses by removing all responsibility for providing verbal study information to patients.

A significant barrier appeared to arise when specialist nurses felt they did not understand the study to their satisfaction, irrespective of its design or focus. This supports previous findings, for example Cvijovic and colleagues’ study, which found that pharmacists were reluctant to invite patients when they felt this could prompt questions they could not answer [10]. Nonetheless, specialist nurses had differing requirements for levels of understanding, and varying levels of prior knowledge and experience to support their understanding. This suggests it could be useful for research teams to tailor the support provided to inviters to enhance understanding.

### Individual (specialist nurse) factors

This study highlights important facilitators stemming from the attitudes, experience, skills and knowledge that specialist nurses bring to the invitation process.

Some specialist nurses held attitudes that they should not apply their own judgements about patient suitability for research studies. Similarly, some described strategies to invite patients in a manner that minimized anticipated negative emotions for patients, involving timing, interpersonal skills and the established clinical relationship. Holding these attitudes, and being willing and able to use these strategies, appeared to be important facilitators. This provides some new insights, as although it has been previously identified that certain clinicians feel all eligible patients should be invited to studies [9, 13], these attitudes have been little explored. It would be beneficial to explore these attitudes and strategies in future research, in order to inform development of recruiter training as advocated by Donovan and colleagues [30], and to support and encourage other clinicians inviting patients to studies.

Specialist nurses appeared to recognise and capitalise on facilitators within their roles because they regarded inviting patients to research as relevant and important. Finding ways to invoke similar positive attitudes in other clinicians could therefore be useful. As identified by previous studies [5, 12], several participants felt that personal research knowledge and experience could invoke research appreciation. It is likely to therefore be useful to increase clinician exposure to and understanding of all aspects of research. Such measures may also support understanding of studies, further facilitating invitations. However, as some participants felt that research knowledge may increase their caution about inviting certain patients, any educational interventions should include consideration of gatekeeping issues.

### Clinical setting factors

A positive research culture appeared to be an important facilitator to specialist nurses inviting patients to participate. Previous studies have found that positive organizational research cultures facilitate recruitment, provided that these result in sufficient provision of research infrastructure [33], and that these values are communicated to staff [7, 15]. The wider organisational research culture within NHS Trusts and clinical specialties was considered potentially influential by some participants. This supports the continuation of national initiatives to raise awareness of and promote involvement with research, for example the *Ok to ask* campaign within the NHS [34].

This study however highlights more strongly the importance of positive research cultures at the very local clinical team level, and provides some insights into how these may be achieved. Many examples of positive cultures appeared to have evolved within clinical teams according to local need and context, suggesting that encouraging local efforts to establish favourable research environments may be most successful. It may also be helpful to explore the development and features of established positive local research cultures in future research.

### Strengths and limitations

Despite elements of convenience sampling, the final sample of specialist nurses represented a wide variety of clinical specialties and NHS Trusts, who had collective experience of inviting a variety of patient populations to numerous different research studies. This may increase the relevance of findings to a wide variety of other contexts. Nonetheless, more variation in clinical specialties, studies and Trusts may have further increased the range of perspectives obtained. Similarly, although data saturation was not a methodological aim of this study, new ideas continued to emerge during final interviews. This highlights that this study provides only limited insight into the perceptions of the wider population of specialist nurses.

All of the participants in this study expressed positivity towards research, and many had wider experience of research, meaning perceptions of specialist nurses who felt negatively or ambivalent were not obtained. These imbalances are very likely due to the sample being self-selecting, with participation in this study likely being most appealing to specialist nurses with an interest in research. While this is a limitation, several of the insights offered by this study relate to facilitators brought about by positive attitudes and research interest of specialist nurses, affording the opportunity to examine facilitators in more depth than many previous studies.

All participants were female and many had higher educational qualifications. This may again have limited the breadth of views obtained, however is likely reflective of the demographics of the specialist nurse workforce in the NHS.

A key strength of the data collection method is that the interviewer was not linked to the research studies or research teams discussed by participants, which may have encouraged participants to give more open and honest responses. The Framework data analysis method provided a clear audit trail to demonstrate how study findings developed from the original data [35].

We could have strengthened the study by using an established theoretical framework to guide data collection and analysis. For example, the Theoretical Domains Framework (TDF) [36, 37], would have enabled us to explore healthcare professional behaviour more systematically. The five themes our study has identified do seem to map well to five of the TDF domains. Assessing patient suitability resembles TDF’s *beliefs about consequences*; team work shares similarities with s*ocial/professional role and identity*, valuing research with *motivation and goals*, invitation process with e*nvironmental context and resources*, and understanding the study with k*nowledge and skills*. Nonetheless, using the TDF during the study would have prompted us to ask additional questions relating to the other domains, which would likely have enriched the findings.

Some novel insights were offered by specialist nurses in this study. Certain issues may relate more to specialist nurses than other healthcare professionals, suggesting caution is necessary if applying findings beyond this professional group. Nevertheless, the detailed nature of the findings in this study aids those considering findings and recommendations to judge their wider applicability.

## Conclusion

This study provides some important insights into facilitators, which appeared to help specialist nurses overcome barriers relating to time, workload and organisational issues reported in some previous studies. These facilitators often stemmed from specialist nurses’ attitudes, skills and experience, and similarly from positive research cultures and teamwork at the local level. As we could only explore these facilitators superficially in this broadly-focused study, we recommend further qualitative research to explore how these develop and can be sustained.

In contrast to some previous studies, specialist nurses generally wanted to spend time explaining studies to patients, and they generally required a good level understanding of studies. Research teams should take this into account when collaborating with specialist nurses and ensure they understand individual needs. Positive relationships and effective communication between research teams and specialist nurses are vital to achieving this, and indeed relationships and communication were key factors across all themes. This resonates with many previous findings, further stressing the importance of mutual engagement and collaboration between research and clinical teams to successful study recruitment.

The increased knowledge and understanding of barriers and facilitators faced by specialist nurses inviting patients to participate in research provides evidence relevant to all stakeholders with an interest in increasing patient research participation. These stakeholders are likely to include research teams, specialist nurses and other healthcare professionals, and policymakers, all of whom may reflect on these findings and decide how they may be applicable to local practice circumstances. Findings may also inform the development of recruitment interventions to be evaluated in future research.

## Abbreviations

NHS, National Health Service; RCT, Randomised Controlled Trial; TDF, Theoretical Domains Framework

## Additional file

Additional file 1: Interview Topic Guide contains the topic guide used to conduct the interviews. (PDF 73 kb)
