# Supplementary material for: Specialist nurses’ perceptions of inviting patients to participate in clinical research studies: a qualitative descriptive study of barriers and facilitators
Source: BMC Med Res Methodol. 2016 Aug 11;16:96. doi: 10.1186/s12874-016-0204-5 (PMC4982234; doi:10.1186/s12874-016-0204-5)
Supplement: Additional file 1: — Interview Topic Guide contains the topic guide used to conduct the interviews. (PDF 73 kb) [file 12874_2016_204_MOESM1_ESM.pdf]

Additional file 1 - Interview Topic Guide

Introductory Questions

- Please tell me your job title and a bit about your job.

- This interview is about your experiences of inviting patients to take part in research studies. Can you tell me a little bit about the studies you're involved with?
    - Prompt: How do you invite patients to take part?
    - Prompt: How did you get involved in these studies?
    - Prompt: What kind of studies are they?

- Have you had any previous experience of research?
    - Prompt: In a job, as a student or other?

- How do you feel about clinical research generally?

Main Questions

- When you're seeing a patient what kinds of things do you think make it more likely that you will invite him or her to take part in a research study, or what kinds of things do you think enable you to do that?

- What kinds of things make it more difficult for you to invite him or her to take part in a research study or less likely that you will?

Ask participants about their experiences of barriers and facilitators in relation to the following:

- Research studies themselves
- The research teams you work with
- Anything about the individual patient
    - **Additional Prompt:** What would make you think that (that they wouldn't be interested etc?)
    - **Additional Prompt:** the relationship you have with the patient
- Nature and setting of your clinical work – how, when, where see patients, organisational factors
- Your own opinions or knowledge or experience of research / in general
- Anything else

Closing Question

- Is there anything else you've thought of that you would like to mention?
